# Behind Closed Doors: Elder Abuse as an Overlooked Pandemic of Modern Times

**DOI:** 10.3390/nursrep16070249

**Published:** 2026-07-17

**Authors:** Karolina Filipska-Blejder, Monika Biercewicz, Renata Jabłońska, Agnieszka Królikowska, Beata Haor, Robert Ślusarz

**Affiliations:** 1 Neurological and Neurosurgical Nursing Department, Faculty of Health Science, Collegium Medicum in Bydgoszcz, Nicolaus Copernicus University in Toruń, Łukasiewicza 1 Street, 85-821 Bydgoszcz, Poland; renata.jablonska@cm.umk.pl (R.J.); agnieszka.k.75@cm.umk.pl (A.K.); beata.haor@cm.umk.pl (B.H.); robert_slu_cmumk@wp.pl (R.Ś.); 2 Clinic of Geriatrics, Faculty of Health Science, Collegium Medicum in Bydgoszcz, Nicolaus Copernicus University in Toruń, Skłodowskiej 9 Street, 85-094 Bydgoszcz, Poland; monika_bierc_cmumk@wp.pl

**Keywords:** elder abuse, older adults, review, risk factors, COVID-19

## Abstract

Elder abuse is internationally recognized as a growing problem of the 21st century that requires urgent intervention and action. This review aimed to synthesize evidence on the prevalence and risk factors of elder abuse between 2010 and 2022. A total of 2875 articles were identified through database searches, of which 28 met the inclusion criteria and were included in the final analysis (24 cross-sectional studies, 3 prospective studies, and 1 descriptive study). Reported prevalence rates of elder abuse ranged from 2.2% to 81.2% overall, while studies conducted during the COVID-19 pandemic reported rates ranging from 1.6% to 44.7%. Psychological abuse was the most frequently reported form of violence. The most commonly identified risk factors included low income, low educational level, gender, disability, depression, and—during the COVID-19 pandemic—social isolation. Across most studies, individuals with lower education and women were statistically more likely to experience abuse. The findings indicate substantial variability in the prevalence of elder abuse both before and during the pandemic, with consistently high rates observed. These results highlight the urgent need for improved screening, education, and targeted interventions by healthcare and social service professionals.

## 1. Introduction

The World Health Organization (WHO) defines elder abuse (EA) as “a single or repeated act, or lack of appropriate action, occurring within any relationship in which there is an expectation of trust, that causes harm or distress to an older person” [1]. Despite increasing recognition of EA as a major public health and social issue, its conceptualization and interpretation remain inconsistent across the literature. Several theoretical approaches have been proposed to explain EA, including social learning theory, caregiver psychopathology theory, dyadic discord theory, and caregiver stress theory. Social learning theory suggests that individuals exposed to violence during childhood may be more likely to perpetuate violent behaviors later in life. In contrast, caregiver psychopathology theory emphasizes the role of mental disorders and psychological dysfunction in the development of abusive behaviors. Dyadic discord theory highlights the importance of interpersonal conflict, dissatisfaction, and contextual relationship factors, whereas caregiver stress theory focuses on caregiver burden and the inability to adequately cope with caregiving responsibilities. Although these theories contribute to understanding EA, they also present important limitations. Most approaches conceptualize EA as a relatively uniform phenomenon and rarely distinguish between different subtypes of abuse, such as psychological, physical, sexual, or financial abuse. Consequently, they often assume that similar mechanisms underlie all forms of EA, despite evidence suggesting that different forms of abuse may involve distinct risk factors, motivations, and social contexts. Furthermore, many theoretical models predominantly emphasize physical dependency and functional impairment while paying less attention to broader psychosocial, cultural, and structural determinants of abuse [2]. More recently, the socio-ecological framework has been increasingly applied to the study of elder abuse. This model conceptualizes EA as the result of interactions between individual, interpersonal, community, and societal factors, recognizing that abuse is influenced not only by characteristics of older adults and caregivers but also by broader social, cultural, legal, and healthcare environments. Consequently, understanding EA requires consideration of determinants operating across multiple levels rather than focusing exclusively on individual or caregiver-related factors. This may partially explain why research findings on EA remain inconsistent and fragmented across studies. The literature distinguishes several forms of violence, including psychological, physical, economic, and sexual abuse, as well as neglect and EA. Violence constitutes a violation of the fundamental right to freedom and security and requires urgent social and, in some cases, medical intervention [1,2,3,4]. EA is also a significant public health issue, as it has substantial negative consequences for health and well-being from a biopsychosocial perspective. Previous studies have demonstrated that exposure to violence is associated with depression and psychological distress [4,5]. Other studies have shown that EA is associated with increased mortality among older adults [6]. Victims of EA often require hospitalization due to abuse-related injuries [7,8]. Furthermore, experiencing EA reduces subjective well-being and may be associated with a lower quality of life [9,10]. As the global population of older adults increases, EA is becoming an increasingly serious social problem.

The WHO predicts that the number of older adults will reach 1.4 billion by 2030 and 2.1 billion by 2050, representing the fastest growth of any age group [11]. Consequently, since the beginning of the twenty-first century, growing attention has been devoted to the issue of EA. Abuse of older adults is now recognized as a global social, health, and economic problem [1,12,13]. Older adults are at increased risk of EA for several reasons. Compared to younger age groups, older adults are more likely to become financially, emotionally, and physically dependent on others. They also report higher rates of depression, cognitive impairment, and social isolation, which often result in hospitalization and institutionalization [14,15]. These factors significantly increase vulnerability to EA, highlighting the importance of conducting research in this population [12]. Storey [15] reviewed the literature and identified risk factors associated with both victims and perpetrators of EA. Shared risk factors included poor physical and mental health, substance use disorders, dependency, impaired stress management and coping abilities, dysfunctional attitudes, previous victimization, and problematic interpersonal relationships. Similarly, Pillemer et al. [16] identified distinct risk factors for victims and perpetrators. Victim-related factors included functional dependence, poor physical and mental health, cognitive impairment, and low income, whereas perpetrator-related factors included mental illness, substance use, and dependency on the older adult. Research on EA is characterized by substantial discrepancies in reported findings, which considerably limits the comparability and interpretation of existing evidence. The literature points to several explanations for these inconsistencies, including methodological heterogeneity, cultural differences, variations in definitions of EA, and the use of diverse assessment tools and measurement instruments [17]. As a result, prevalence estimates reported across studies vary significantly, making it difficult to determine the actual scale of the problem. Another important issue is the inconsistency in the identification of risk factors associated with EA. Previous reviews have often reported contradictory findings regarding the role of demographic, psychological, social, and economic determinants. Moreover, many studies focus primarily on selected forms of abuse or specific populations, limiting the generalizability of findings. Existing reviews also frequently lack critical discussion regarding methodological limitations, practical implications, and the broader social context of EA. Consequently, despite the growing number of publications on this topic, substantial gaps remain in understanding the prevalence, characteristics, and mechanisms underlying EA. To date, relatively few studies have attempted to critically synthesize findings on EA over an extended period while simultaneously considering the impact of the COVID-19 pandemic. This represents an important gap in the literature, particularly given that the pandemic substantially altered social functioning, healthcare access, caregiving conditions, and interpersonal relationships. Experts from the WHO and the United Nations have warned that the COVID-19 pandemic may have intensified the risk of EA [1,18,19]. Factors such as social isolation, loneliness, financial instability, caregiver burden, reduced access to social support, and increased psychological stress are considered particularly important contributors to violence during public health crises. Existing evidence suggests not only an increase in the prevalence of EA during the pandemic but also possible changes in the characteristics and severity of abuse [20,21]. However, the available findings remain fragmented, and many studies differ substantially in methodology, population characteristics, and definitions of abuse. Therefore, a critical synthesis of the available evidence is needed to better understand the prevalence, forms, and risk factors of EA before and during the COVID-19 pandemic while considering the methodological, cultural, and broader socio-ecological factors that may contribute to variability across studies. Such an approach may also provide a stronger basis for informing public health policies, prevention strategies, and the development of healthcare and social support services for older adults.

Accordingly, this integrative review was undertaken to address these issues and provide a comprehensive synthesis of the available evidence on elder abuse. The review presents worldwide research on the prevalence, characteristics, and risk factors of EA published between 2010 and 2022, encompassing both the pre-pandemic and COVID-19 pandemic periods. An important strength of the review is its comprehensive overview of EA across diverse geographical regions and healthcare contexts. Furthermore, it facilitates a better understanding of discrepancies observed in previous findings by examining methodological, demographic, and contextual factors that may contribute to variability in reported prevalence estimates. Another notable strength is the separate analysis of studies conducted during the COVID-19 pandemic, which allows for a more focused examination of factors associated with EA during this unique period. The title “The Overlooked Pandemic” reflects not only the widespread occurrence of elder abuse across countries and settings but also its persistent under-recognition and the need for stronger public health, social, and policy responses.

## 2. Materials and Methods

### 2.1. Search Strategy

This study was conducted as an integrative review following the methodological framework proposed by Whittemore and Knafl [22]. The conduct and reporting of the review were guided by the Preferred Reporting Items for Systematic Reviews and Meta-Analyses (PRISMA) statement [23] to enhance transparency in the literature search, screening, and study selection processes. The search protocol was not registered. A multidisciplinary team of experts with experience in conducting reviews and methodological expertise systematically reviewed the literature using the following databases: PubMed, Scopus. As part of the search strategy, Google Scholar was used as an additional source to support identification of potentially relevant literature and to enhance the breadth of the database search. The following keywords were used: elder abuse, elder neglect, violence, financial exploitation, physical abuse, psychological abuse, sexual abuse, prevalence of elder abuse, older adults, risk factors, perpetrators, victims, and COVID-19. Detailed search strategies were adapted to the indexing systems and search interfaces of the individual databases. Searches combined terms related to EA, older adults, prevalence, and risk factors using Boolean operators (“AND”, “OR”). The complete search strategies used for each database are provided in Supplementary Table S1. For studies examining EA during the COVID-19 pandemic, the primary search strategy was supplemented with the terms “COVID-19”, “pandemic”, and “coronavirus”. In accordance with WHO guidance, the COVID-19 pandemic period was considered to begin on 11 March 2020 [24]. Studies published between 2021 and 2022 that explicitly reported data collected during the pandemic period were eligible for inclusion. Publications released after 2022 were not included in the review in order to maintain temporal homogeneity of the analyzed evidence and to avoid potential confounding introduced by post-pandemic contextual changes. In particular, more recent studies may reflect additional societal and economic determinants emerging after the acute phase of the COVID-19 pandemic, such as inflationary pressures, prolonged social isolation effects, and broader post-pandemic socioeconomic instability, which could have influenced the prevalence and risk profiles of EA in ways not directly comparable to the pandemic-period evidence base. The literature search, screening, full-text assessment, and manuscript revision processes were conducted between April 2023 and December 2025, while only studies published between 2010 and 2022 that met the predefined eligibility criteria were included in the final review. Accordingly, the present integrative review should be interpreted as a synthesis of evidence published between 2010 and 2022 rather than as a comprehensive representation of the current state of knowledge regarding EA.

### 2.2. Study Selection and Eligibility Criteria

A total of 2875 articles were identified in this category. After the screening process, 28 articles were ultimately included in the review. The review process began with the analysis and evaluation of the titles and abstracts of all retrieved articles by two independent researchers (the screening process was fully double-blind). Duplicate records were removed. The remaining articles were then independently screened for relevance by four researchers. The literature search was limited to articles published in English and reporting results exclusively related to studies involving older adults. Articles that were deemed irrelevant to the aim of this review were excluded. The next stage involved a full-text analysis of the selected articles, conducted by the same two independent researchers. Inclusion criteria were developed a priori using the Population–Concept–Context (PCC) framework to ensure systematic and reproducible study selection. The following eligibility criteria were applied: (i) Population: older adults (aged ≥60 years), including community-dwelling and/or institutionalized populations; (ii) Concept: prevalence, forms, characteristics, and risk factors of elder abuse (EA), including physical, psychological, and sexual abuse, neglect, and financial exploitation; (iii) Context: studies conducted before and/or during the COVID-19 pandemic period; (iv) Study Design: observational studies (cross-sectional, descriptive, and prospective studies). Studies published in English between 2010 and 2022 were included. This language restriction may have introduced language bias and resulted in the exclusion of potentially relevant studies published in other languages. Therefore, the findings should be interpreted with caution, particularly with regard to their generalisability across different cultural and geographical contexts. This limitation is discussed further in Section 5, Limitations.

The exclusion criteria were as follows:

(1) studies focusing exclusively on populations defined by a specific disease entity (e.g., cardiovascular diseases, cancer);
(2) studies in which the analysis concerned only women or only men, or populations younger than 60 years.

### 2.3. Critical Appraisal

To support interpretation of the included evidence, a simplified methodological appraisal informed by selected domains of the Joanna Briggs Institute (JBI) critical appraisal framework for observational studies was conducted. The assessment considered study design, sample size, measurement approach, use of validated instruments, and reporting of statistical analyses. The appraisal was intended to provide a general overview of the methodological characteristics of the included studies and to facilitate interpretation of the findings rather than to provide a comprehensive study-level assessment of methodological quality or risk of bias. The results of this assessment are presented in Supplementary Table S3. No studies were excluded on the basis of this appraisal, as the objective was to provide a comprehensive synthesis of the available evidence and to contextualize the methodological strengths and limitations of the included studies. The results of the appraisal were used to support interpretation of the findings and to contextualize the methodological strengths and limitations of the included studies rather than to weight the evidence or exclude studies.

### 2.4. Data Extraction

The following data were collected from the qualified studies: (1) name and surname of the authors, (2) year of publication, (3) study project, (4) study objectives, (5) characteristics of the studied population, (6) study setting, (7) methods of assessing the occurrence of violence and its risk factors, (8) type(s) of violence used, (9) detailed characteristics of risk factors, including behavioral, environmental, comorbidity-related, demographic, family-related, and individual factors.

A quantitative meta-analysis was not performed because of substantial methodological heterogeneity across the included studies. The studies differed considerably with respect to operational definitions of EA, study populations, research settings, assessment instruments, outcome measures, and methodological designs. Furthermore, significant variability was observed in the reporting of prevalence estimates and risk factors, limiting the comparability of findings across studies. Therefore, conducting a quantitative meta-analysis was considered methodologically inappropriate and potentially misleading. Therefore, an integrative review was considered more suitable to provide a comprehensive and context-sensitive synthesis of the available evidence.

## 3. Results

### 3.1. Overview of Studies

**Figure 1:**
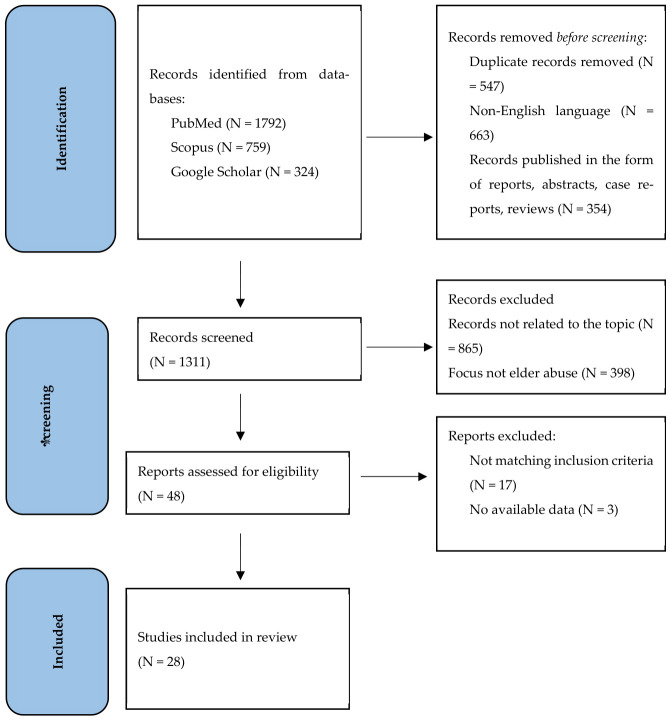
PRISMA flow diagram of the literature search strategy.

A total of 2875 articles were identified through the database search. After the removal of duplicates (n = 547), the titles and abstracts were screened, and 48 articles were selected for full-text review. Ultimately, 28 articles were included in the final analysis. Figure 1 presents the PRISMA flow of the review process. Of the 28 included studies, 25 were cross-sectional [20,21,24,25,26,27,28,29,30,31,32,33,34,35,36,37,38,39,40,41,42,43,44,45,46,47], one was a descriptive study [48], and two were prospective studies [49,50]. The studies included in this review were published between 2010 and 2022. Sample sizes ranged from 146 participants [32] to 18,236 participants [38]. All studies included both men and women; however, women constituted the majority of participants in most samples. The study populations consisted exclusively of older adults. In most studies, participants were aged 60 years and older [20,25,26,27,28,30,31,32,34,35,36,37,39,40,41,42,45,46,47,50]. In the remaining studies, the minimum age criterion was 65 years [21,29,33,38,43,44,49]. Detailed characteristics of the included studies are presented in Table 1.

### 3.2. Assessment and Tools Used

Across the included studies, the prevalence of EA ranged from 2.2% of respondents [40] to 81.2% [27]. The substantial heterogeneity in reported prevalence estimates may be attributable to several methodological and contextual factors across the included studies. Considerable variability was observed in the operational definitions and measurement of EA, with some studies assessing overall exposure to violence and others focusing on specific forms of abuse, including psychological, physical, sexual, or financial abuse. Furthermore, the studies were conducted across diverse cultural, socioeconomic, and healthcare contexts, which may have influenced both the occurrence, recognition, and reporting of abuse. Additional heterogeneity stemmed from differences in the characteristics of the investigated populations, including age distribution, health status, living arrangements, degree of functional dependence, and caregiving circumstances. The included studies also differed with respect to assessment instruments, data collection procedures, and study settings, encompassing community-based samples, clinical populations, hospital settings, and shelter homes. Collectively, these methodological and contextual differences may have substantially influenced prevalence estimates and limit the direct comparability of findings across studies. Psychological abuse was the most frequently reported form of violence, identified in 17 studies [20,21,26,27,28,29,30,31,34,35,36,39,42,43,44,46,50]. This was followed by economic abuse, reported in two studies [40,48], and neglect, identified in two studies [25,33] (Table 2).

A variety of instruments were used to assess the prevalence of EA. The most frequently applied tools were author-designed questionnaires [20,21,26,27,34,35,39,43], the Hwalek–Sengstock Elder Abuse Screening Test (H-S/EAST) [32,34,37,46], and the Conflict Tactics Scale (CTS) [30,40,47]. In eleven studies, data were collected through home-based interviews [25,28,30,31,36,40,41,45,46,47,49], while one study conducted interviews in a community setting [42]. Several studies were conducted in hospital settings [21,32,34,35,44,50], whereas others were carried out in health centers [37,39]. Data were also collected via online surveys [20,26,38,43]. These methodological differences may have influenced disclosure rates and contributed to the observed variability in EA prevalence (Table 1).

### 3.3. Risk Factors

Risk factors for EA are summarized in Table 2 and Table S4. Across the included studies, economic difficulties emerged as the most consistently reported risk factor and were identified in 19 studies [20,21,25,26,27,28,30,31,32,35,36,37,39,40,41,42,46,47,50]. The findings suggest that financial instability was frequently associated with EA across the included studies. Financial difficulties were often linked to caregiver burden, dependence, and interpersonal stress, although causal relationships could not be established. Age was also associated with the occurrence of EA. However, for this risk factor, it is not possible to precisely determine which age categories are at the highest risk, as the findings across studies are inconsistent [25,27,28,30,32,35,36,37,39,42]. For example, Dos Santos et al. [32] reported that nearly 81% of individuals aged over 90 years experienced violence. In contrast, Anand [28] indicated that individuals aged 80 years and older were statistically more vulnerable to abuse than younger age groups, whereas Chokkanathan [30] identified individuals aged 70 years and older as being at increased risk. These inconsistencies may reflect differences in study populations, definitions of EA, and analytical approaches. Education emerged as a significant risk factor in ten studies [26,28,29,33,36,37,39,41,44,46]. Gender was also identified as a significant risk factor [25,26,27,30,31,33,35,42,44], as was disability [25,28,30,31,36,41,42,46]. In the majority of studies, lower educational attainment and female sex were associated with a higher reported occurrence of EA. Another important risk factor for EA was depression, which was identified in several studies [26,29,42,45,46,50]. Although these variables were frequently associated with EA, the findings should be interpreted cautiously. The included studies differed substantially in methodological quality, adjustment for confounding variables, and statistical approaches. Consequently, the observed associations should not be interpreted as evidence of direct causality or as indicators of the relative strength of evidence for individual risk factors, but rather as patterns of vulnerability reported across heterogeneous studies.

### 3.4. Elder Abuse During COVID-19 Pandemic

Studies conducted during the COVID-19 pandemic reported highly heterogeneous prevalence estimates of EA, ranging from 1.6% [38] to 44.7% [21] (Table 2). Despite this variability, several investigations reported higher levels of specific forms of abuse among older adults during the pandemic period.

Weissberger et al. [48] reported an increase in psychological abuse (from 28.9% to 35.8%) and physical abuse (from 6.4% to 14.4%) during the pandemic. Similarly, Chang and Levy [20] observed increases in financial abuse (from 3.5% to 7.5%) and physical abuse (from 1.6% to 5.4%). Filipska et al. [21] demonstrated that nearly 45% of older adults experienced some form of violence during the COVID-19 pandemic, compared with 38.5% reported in the same population before the pandemic [35]. Together, these findings suggest that higher rates of EA and specific forms of abuse were reported in several studies conducted during the pandemic period. However, given the observational nature of the available evidence, these findings should be interpreted as associations rather than evidence of a direct effect of the pandemic on EA occurrence.

One of the most consistently reported pandemic-related factors was social isolation. Public health measures such as lockdowns, physical distancing, and reduced access to social and healthcare services were frequently discussed in the included studies as factors potentially associated with increased social isolation and reduced access to support systems among older adults. Koga et al. [38] reported that reduced interaction with neighbors was associated with an increased risk of EA (OR 1.37; 95% CI 1.04–1.81). Similarly, Son and Cho [43] identified COVID-19-related social isolation as a significant risk factor (OR 1.47; 95% CI 1.07–2.01).

Additionally, COVID-19 infection was identified in one study as a factor associated with increased vulnerability among older adults [21]. Several studies have reported associations between chronic illness, physical dependence, institutionalization, depressive symptoms, anxiety, and EA. Consequently, COVID-19-related health complications may represent an important area for future investigation; however, causal pathways cannot be determined based on the studies included in this review. Economic instability during the pandemic period was frequently discussed in the included studies alongside caregiver burden and household stress. However, the available evidence does not permit conclusions regarding the direction or causality of these relationships.

Although several studies reported higher levels of EA or factors associated with EA during the pandemic period, direct comparison of findings remains challenging because of differences in study design, definitions of EA, measurement tools, and population characteristics. Therefore, the findings should be interpreted as associations observed in the reviewed studies and as indications of broader patterns reported during the pandemic period rather than as evidence of causal relationships or precise epidemiological estimates.

**Table 2:** Prevalence of elder abuse and its main risk factors.

Author, Year	Prevalence	Type of Elder Abuse	Key Finding of Risk Factors
***During the COVID-19 pandemic***			
Koga et al., 2022 [38]	1.6%	----	*Behavioral factors:* reduced grocery shopping—OR 1.60 (95%CI 1.20–2.13) * Environmental factors: reduced interaction with neighbors—OR 1.37 (95%CI 1.04–1.81) *
Son and Cho, 2022 [43]	8.6%	psychological—83.5% neglect—6.4% physical—5.9% financial—4.2%	*Behavioral factors:* smoking—OR 1.7 (95CI 1.13–2.56) * frequency of None 1 alcohol consumption—1–3 times/month—OR 0.54 (95%CI 0.34–0.84) * Comorbidities: cognitive impairment—OR 5.79 (95%CI 3.34–10.05) *** Demographic factors: age >75 vs. 65–74—OR 0.64 (95%CI 0.45–0.9) * Environmental factors: social isolation due to COVID-19—OR 1.47 (95%CI 1.07–2.01) *
Weissberger et al., 2022 [48]	---	Time 1: Financial—45.8% Psychological—28.9% Neglect—22.1% Physical—6.4% Time 2: Financial—41.1% Psychological—35.8% Neglect—21.3% Physical—14.4%	---
Du and Chen, 2021 [33]	15.4%	neglect—43.7%, financial—40.5% psychological—25.8%, physical—10.2%,	**Neglect:** *Demographic factors:* 70–74 vs. 65–69 years- B—0.18 **, OR 1.25 (95%CI 0.35–2.82) ≥75 vs. 65–69 years- B—0.13 **, OR 1.56 (95%CI 0.67–3.54) Female- B—0.39 **, OR 1.87 (95%CI 0.76–2.18) No religious belief-B—−0.26 **, OR 0.77 (95%CI 0.11–1.64) *Education:* Primary school to high school vs. Illiterate-B—−0.26 **, OR 0.82 (95%CI 0.15–1.34) University and above vs. Illiterate- B—−0.23 *, OR 0.43 (95%CI 0.02–1.83) *Family risk factors:* Number of children 1–2 vs. 0- B—0.31 *, OR 1.52 (95%CI 0.63–2.18) Number of children 3 vs. 0- B—0.21 *, OR 1.55 (95%CI 0.37–2.16) Widowed vs. Married- B—0.42 *, OR 1.86 (95%CI 0.56–3.15) **Financial abuse:** *Education:* Primary school to high school vs. Illiterate- B—−0.21 **, OR 0.54 (95%CI 0.12–2.13) University and above vs. Illiterate- B—−0.54 **, OR 0.83 (95%CI 0.27–1.67) *Family risk factors:* Number of children 1–2 vs. 0- B—0.11 *, OR 1.23 (95%CI 0.87–3.18) Number of children 3 vs. 0- B—0.34 *, OR 1.67 (95%CI 0.54–1.65) **Psychological abuse:** *Demographic factors:* Female- B—0.31 *, OR 1.15 (95%CI 0.76–1.86) No religious belief- B—0.75 **, OR 2.54 (95%CI 0.67–4.32) **Physical abuse:** *Family risk factors:* Number of children 1–2 vs. 0- B—0.21 *, OR 1.12 (95%CI 0.87–1.29) Number of children 3 vs. 0- B—0.16 *, OR 1.08 (95%CI 0.96–1.75)
Chang and Levy, 2021 [20]	21.3%	Before COVID-19: Psychological—9.0% Financial—3.5% Physical—1.6% During COVID-19: Psychological—9.2% Financial—7.5% Physical—5.4%	*Demographic factors:* age 70–79 vs. 60–69—OR 0.67 (95%CI 0.45–0.99) * Family risk factors: sense of community—OR 0.89 (95%CI 0.86–0.93) *** Individual risk factors: financial strain—OR 1.08 (95%CI 1.02–1.14) * physical distancing—OR 0.94 (95%CI 0.89–0.98) **
Filipska et al., 2021 [21]	44.7%	psychological—72.3% neglect—61.9% physical—39.4% financial—36.8% sexual—12.9%	*Comorbidities:* severe depression—OR 18.49 (95%CI 3.91–87.30) *** severe functional impairment—OR 21.07 (95%CI 7.12–62.35) *** COVID-19 in the past—OR 1.59 (95%CI 1.03–2.46) * Demographic factors: age 60–65 vs. >70 years—OR 2.35 (95%CI 1.28–4.31) * age 66–70 vs. >70 years—OR 1.98 (95%CI 1.05–3.75) * female—OR 1.90 (95%CI 1.23–2.93) * lives in an urban area—OR 2.06 (95%CI 1.28–3.31) * Family risk factors: poor relationship with the family—OR 3.26 (95%CI 1.96–5.43) *** Individual risk factors: low equivalent family income vs. high—OR 3.60 (95%CI 1.93–6.72) *** very often or almost always lonely—OR 2.89 (95CI 1.46–5.72) *
***Before the COVID-19 pandemic***			
Ahnlund et al., 2020 [26]	4.2%	psychological—2%, sexual—2%, physical—0.8%	*Education:* lower-level education—OR 0.61 (95%CI 0.39, 0.94) * Individual risk factors: economic problems—OR 3.54 (95%CI 2.04, 6.12) *** retired pensioner—OR 0.55 (95%CI 0.37, 0.81) * Comorbidities: poor physical health—OR 1.91 (95%CI 1.27, 2.81) *** poor psychological health—OR 4.74 (95%CI 3.17, 7.10) *** disability—OR 1.83 (95%CI 1.13, 2.98) * PTSD symptoms—OR 5.73 (95%CI 3.09, 10.62) *** depressive symptoms—OR 2.14 (95%CI 1.14, 4.00) *
Alraddadi, 2020 [27]	81.2%	psychological—71%, neglect—67%, financial—54%, physical—13%	**Psychological abuse:** *Demographic factors:* female—OR 4.70 (95%CI 2.55, 8.83) *** Neglect: *Comorbidities:* chronic disease—OR 2.12 (95%CI 1.33, 3.38) * Demographic factors: female—OR 3.13 (95%CI 1.78, 5.49) *** Individual risk factors: having own income—OR 0.65(95%CI 0.42, 0.99) * Physical abuse: *Comorbidities:* chronic disease—OR 7.32 (95%CI 2.19, 24.46) * Demographic factors: age 70–74 (reference to 60–64)—OR 0.15 (95%CI 0.03, 0.65) * Family risk factors: widow (reference to married)—OR 2.69 (95%CI 1.16, 6.26) *** single—OR 6.10 (95%CI 1.78, 20.94) * Financial exploitation: *Comorbidities:* chronic disease—OR 2.14 (95%CI 1.33, 3.46) * Demographic factors: female—OR 6.29 (95%CI 3.18, 12.44) *** Family risk factors: having children—OR 2.58 (95%CI 1.36, 4.88) *
Dos Santos et al., 2020 [32]	69.9%	N/A	*Demographic factors:* age >90–80.7% *** Family risk factors: more than six children—80.8% * Individual risk factors: frailty syndrome—88.1% *** unemployed—73.7% *
Fang et al., 2020 [50]	50%	psychological—45.3%, physical—13%, multiple—9.3%	*Comorbidities:* cognitiv e impairment—Mean 18.25 (SD 7.57) * depression—N = 116 (38.7%) * neuropsychiatric symptoms—Mean 23.25 (SD 29.87) *
Kulakçi, Altintas, and Korkmaz Aslan, 2020 [39]	13.6%	psychological—11.9%, neglect—3.9%, financial—3.2%, physical—2.7%	**Psychological abuse:** *Demographic factors:* 75 years and older—OR 1.99 (95%CI 1.11, 3.59) * Education: illiterate—OR 4.86 (95%CI 2.81, 8.42) ** Individual risk factors: low income—OR 2.19 (95%CI 1.18, 4.06) * Neglect: *Family risk factors:* bad family relationship—OR 20.56 (95%CI 5.45, 77.55) ** Financial exploitation: *Demographic factors:* 75 years and older—OR 4.37 (95%CI 1.69, 11.28) ** Family risk factors: bad family relationship—OR 27.71 (95%CI 4.66, 164.89) *** Physical abuse: *Individual risk factors:* low income—OR 7.79 (95%CI 2.52–24.13) ***
Sathya and Premkumar, 2020 [41]	11.4%	N/A	*Comorbidities:* 1 disability—OR 1.93 (95%CI 1.59, 2.34) *** 2+ disability—OR 2.47 (95%CI 1.90, 3.21) *** Education: 10+ years of education—OR 0.64 (95%CI 0.48, 0.86) *** Individual risk factors: 2 + ADL—OR 1.31 (95%CI 0.96, 1.80) ** 2 + IADL—OR 2.47 (95%CI 1.90, 3.21) *** higher income (richest)—OR 0.30 (95%CI 0.22, 0.40) ***
Sembiah et al., 2020 [42]	25.6%	psychological—96.8%, neglect—95.2%, financial exploitation—30.1%, physical—30.1%	*Comorbidities:* >2 chronic illness—OR 3.2 (95%CI 1.6, 6.4) *** depression—OR 3.2 (95%CI 1.7, 5.8) *** Demographic factors: age > 70—OR 3.9 (95%CI 2.1–7.3) *** female—OR 2.2 (95%CI 1.2, 4.0) ** Family risk factors: nuclear type of family—OR 1.8 (95%CI 1.1, 3.4) * number of children>3—OR 2.4 (95%CI 1.3, 4.4) ** Individual risk factors: ADL dependent—OR 3.8 (95%CI 2.1- 6.9) *** IADL dependent—OR 3.4 (95%CI 1.7, 6.8) ***
Sudan et al., 2020 [44]	12.7%	psychological—47.4%, neglect—21.1%, mixed—18.4%, exploitation—10.5%, physical—2.6%	*Comorbidities:* psychiatric illness—N = 32 (21.3%) * somatic illness—N = 6 (4%) * Demographic factors: female—N = 34 (84.2%) * Education: illiterate—N = 29 (73.6%) * Family risk factors: widowed—N = 26 (68.3%) *
Filipska et al., 2019 [35]	38.5%	psychological—75.3%, financial—68.8%, physical—48.1%, sexual—22.1%	**Psychological abuse:** *Demographic factors:* female—OR 1.94 (95%CI 1.02, 3.67) * city—OR 3.23 (95CI 1.63, 6.42) ** Financial abuse: *Demographic factors:* female—OR 1.98 (95%CI 1.02, 3.83) * >70 years—OR 3.83 (95%CI 1.51, 9.72) ** Physical abuse: *Demographic factors:* female—OR 2.48 (95%CI 1.13, 5.44) * >70 years—OR 2.97 (95%CI 1.11, 7.95) * city—OR 2.36 (95%CI 1.07, 5.19) * Sexual abuse: *Demographic factors:* female—OR 4.05 (95%CI 1.13, 14.5) * city—3.87 (95% CI 1.08, 13.94) *
Yunus et al., 2017 [47]	8.1%	financial abuse—4.9% psychological—3.3% physical—1.2% neglect and sexual abuse—0.2%	low income- N=99 (74.7%)* GDS score- Mean 4,82 ± 3,90**
Choi et al., 2018 [29]	21.5%	psychological—18%, financial exploitation—6.4%, neglect—3.5%, physical—2.9%, sexual—1.7%	*Comorbidities:* depression—OR 2.23 (95%CI 1.07, 4.73) * PTSD—OR 4.19 (95%CI 1.98, 9.18) *** Education: high school or above—OR 0.37 (95%CI 0.14, 0.92) * Family risk factors: unmarried/separated/divorced—OR 4.18 (95%CI 1.40, 12.61) **
Torres-Castro et al., 2018 [45]	35.7%	conflict abuse—33.4%, financial exploitation—12.3%, caregiver neglect—6.6%	*Comorbidities:* depression—N = 100 (57.5%) *** Individual risk factors: frailty—N = 30 (17.2%) *** anxiety—N = 101 (58.0%) ***
Hosseinkhan et al., 2017 [37]	38.5%	N/A	*Demographic factors:* age—OR 0.96 (95%CI 0.93, 0.99) * lives in urban area—OR 3.53 (95%CI 1.97, 6.32) *** Education: college vs. illiterate—OR 6.39 (95%CI 1.74, 23.44) ** Individual risk factors: lack of income—OR 1.0 (95%CI 1.0, 1.1) *
Anand, 2016 [28]	35%	verbal—31.9%, disrespect—24.4%, financial exploitation—22.5%, neglect—19.5%, physical—18%	*Demographic factors:* age ≥ 80—OR 1.87 ** Education: >8 years of education—OR 0.33 ** Family risk factors: living with family—OR 0.57 * Individual risk factors: no disability—OR 0.63 ** highest health quintile—OR 0.31 **
Giraldo-Rodríguez et al., 2015 [36]	32.1%	psychological—28.1%, financial exploitation—11.9%, physical—7.0%, sexual—2.5%	*Demographic factors:* 80 and older—OR 1.99 (95%CI 1.31, 3.02) *** lives in urban area—OR 1.52 (95%CI 1.04, 2.20) * Education: >9 years of education—OR 4.98 (95%CI 3.04, 8.17) *** Individual risk factors: unemployed—OR 1.75 (95%CI 1.13, 2.73) **,
Chokkanathan, 2014 [30]	21.0%	psychological—19.2%, financial exploitation—12.7%, neglect—12.4%, physical—12.3%	*Demographic factors:* >70 years—OR 1.45 (95%CI 1.01, 2.09) * male—OR 0.61 (95%CI 0.40–0.92) * Individual risk factors: unemployed—OR 1.52 (95%CI 1.00, 2.32) * dependency—OR 1.14 (95%CI 1.01, 1.28) *
Edirisinghe et al., 2014 [34]	45%	psychological—26%, financial exploitation—22%, verbal—10%, physical—2.6%	*Family risk factors:* currently single—N=107 (20.2%)
Abdel Rahman and El Gaafary, 2012 [25]	43.7%	neglect—42.4%, physical—5.7%, psychological—5.1%, financial exploitation—3.8%	*Demographic factors:* age ≥ 70 years—OR 2.5 (95%CI 1.2–5.2) ** Family risk factors: no. children ≤3—OR 5.08 (95%CI 1.9, 13.4) *** Individual risk factors: insufficient pension—OR 3.3 (95%CI 1.3, 8.6) ** dependency—OR 17.6 (95%CI 5.4, 57.2) ***
Dong et al., 2012 [49]	black: men, 13.2%; women, 10.9% white: men, 2.4%; women, 2.6%	N/A	*Individual risk factors:* lowest tertile of physical performance testing—OR = 4.92 (95%CI = 1.39, 17.46)
Naughton et al., 2012 [40]	2.2%	financial exploitation—61.5%, psychological—56.9%, physical—22.5%, neglect—13.8%, sexual—2.3%	*Comorbidities:* below average of physical health—OR 1.63 (95%CI 0.78, 3.40) below average of mental health—OR 4.51 (95%CI 2.22, 9.14) *Environmental factors:* poor social support—OR 3.11 (95%CI 1.29, 7.46) *Individual risk factors:* lower income—OR 1.64 (95%CI 0.67, 3.95)
Wu et al., 2012 [46]	36.2%	psychological—27.3%, neglect—15.8%, physical—4.9%, financial exploitation—2.0%,	*Comorbidities:* depression—OR 5.5 (95%CI 4.1, 7.3) ** Individual risk factors: physical disability—OR 1.5 (95%CI 1.1, 2.2) * Family risk factors: widowed/divorced/single/separated—OR 1.8 (95%CI 1.4, 2.4) ** living with spouse and children—OR 0.7 (95%CI 0.5, 0.9) *
Chompunud et al., 2010 [31]	14.6%	psychological—41.2%, financial exploitation—20.6%, physical—2.9%, neglect—2.9%	*Demographic factors:* female—OR 5.1 (95%CI 1.3, 18.8) * Family risk factors: high family dependency—OR 6.3 (95%CI 1.1, 35.0) ***

Note. ADL = Activities of Daily Living; B = regression coefficient; CI = confidence interval; GDS = Geriatric Depression Scale; IADL = Instrumental Activities of Daily Living; N/A = not applicable; OPD = Outpatient Department; OR = odds ratio; PTSD = post-traumatic stress disorder; SD = standard deviation. * Significant at *p* < 0.05, ** *p* < 0.01, *** *p* < 0.001.

## 4. Discussion

To our knowledge, this is one of the first reviews comprehensively examining EA across the period spanning both the pre-pandemic and COVID-19 pandemic eras (2010–2022). The review integrates international evidence regarding the prevalence of EA, its forms, and associated risk factors across diverse cultural, healthcare, and socio-ecological contexts. By synthesizing studies conducted in community, clinical, institutional, and shelter-home settings, this review provides a broad overview of factors contributing to vulnerability among older adults.

The findings demonstrate substantial heterogeneity in the reported prevalence of EA, with estimates ranging from 2.2% [40] to 81.2% [27]. This variability is likely attributable to differences in operational definitions of EA, methodological approaches, cultural norms, study populations, assessment instruments, and research settings. A structured overview of these sources of heterogeneity is presented in Supplementary Table S2. Importantly, cultural perceptions regarding family relationships, ageing, and violence may influence not only the occurrence of abuse but also its disclosure and social recognition. Furthermore, studies differed considerably in their sampling strategies, research settings, and methods of data collection, thereby limiting direct comparability of prevalence estimates. Consequently, prevalence findings should be interpreted within the methodological and sociocultural context of individual studies [25,35,50]. Furthermore, because the included studies differed substantially with respect to adjustment for confounding variables, statistical models, and analytical approaches, the frequency with which individual factors were reported should not be interpreted as a direct indicator of the strength of evidence. Accordingly, the identified risk factors should be viewed as patterns of association observed across heterogeneous studies rather than as ranked or causal determinants of elder abuse. The simplified methodological appraisal indicated that many of the reported associations were observed across studies characterized by relatively large sample sizes and appropriate reporting of statistical analyses. However, because the evidence base consisted predominantly of cross-sectional studies with considerable methodological heterogeneity, the findings should be interpreted as consistent patterns of association rather than evidence of causality.

Despite this heterogeneity, several relatively consistent patterns emerged across the reviewed literature. Economic difficulties, disability, depressive symptoms, functional dependence, chronic illness, and social isolation were repeatedly identified as factors associated with EA [25,26,27,28,29,30,31,32,34,35,36,37,39,40,41,42,44,45,46,47,48,49,50]. These findings support broader theoretical perspectives emphasizing the interaction between social vulnerability, dependency, and reduced access to supportive social networks in later life. Older adults with poor physical health, cognitive impairment, and limited socioeconomic resources were more frequently identified in the reviewed studies as populations potentially vulnerable to elder abuse. The present findings may also be interpreted within a broader socio-ecological framework, which conceptualizes elder abuse as the result of interactions between individual, interpersonal, community, and societal determinants. The identified associations with disability, cognitive impairment, depressive symptoms, and functional dependence reflect vulnerabilities at the individual level, whereas caregiver burden, family relationships, and interpersonal conflict correspond to the interpersonal level. Social isolation and reduced community support represent community-level influences, while socioeconomic inequalities, healthcare accessibility, and the availability of formal support services reflect broader societal determinants. These observations support the view that effective prevention of elder abuse requires coordinated interventions across multiple levels, extending beyond approaches focused exclusively on individual risk factors.

The relationship between cognitive impairment, functional decline, and EA deserves particular attention. Cognitive disorders and dementia may reduce an individual’s capacity for self-protection, communication, and independent functioning, thereby increasing dependence on family members or caregivers [26,41,50,51,52]. However, the relationship between caregiver burden and abusive behavior appears to be multidimensional rather than directly causal. Psychological stress, depressive symptoms, financial strain, social isolation, lack of institutional support, and pre-existing family conflict may collectively contribute to caregiver distress and dysfunctional caregiving dynamics. Therefore, caregiver-related risk should be interpreted within a broader psychosocial and socioeconomic context. These observations are also consistent with the conceptual frameworks proposed by Storey [15] and Pillemer et al. [16], which emphasize that elder abuse arises from the interaction of victim, perpetrator, relationship, and environmental factors rather than from vulnerabilities of older adults alone. In addition to caregiver burden, perpetrator-related characteristics such as mental health problems, substance use, dependency on the older adult, and ineffective coping strategies may contribute to abusive behaviours when combined with adverse psychosocial circumstances. Furthermore, community resources, healthcare accessibility, social support services, and legal protection mechanisms may influence both the occurrence and disclosure of elder abuse, supporting the need for prevention strategies that extend beyond the individual and family levels.

Recent evidence further indicates that caregivers of individuals with dementia do not constitute a homogeneous group but may present distinct psychological profiles characterized by varying combinations of anxiety, depressive symptoms, stress, coping strategies, and resilience. These findings suggest the need for individualized psychological assessment and support interventions tailored to caregivers’ specific needs. Moreover, the experience of dementia is influenced not only by cognitive decline itself but also by caregiving practices, socioeconomic limitations, family relationships, and cultural interpretations of illness, all of which may affect the quality of life of both older adults and their caregivers [53].

The reviewed studies also demonstrated significant associations between depressive symptoms and EA. Depression was consistently associated with EA across several studies. However, owing to the predominantly cross-sectional design of the included studies, the temporal direction of this relationship could not be determined. In this context, family climate, communication patterns, cohesion, and marital or interpersonal relationships may play an important protective or harmful role. Previous research suggests that reduced family cohesion, impaired communication, loneliness, and socioeconomic vulnerability are closely associated with poorer mental health outcomes among older adults. These psychosocial factors were frequently discussed in the literature as being associated with increased vulnerability to EA, potentially through reduced access to emotional support and greater dependence on caregivers or family members [54].

Several studies indicated that women were more frequently identified as victims of EA [27,31,35,42,44]. However, these findings should be interpreted cautiously, as gender differences may be influenced by social, cultural, and methodological factors, including differences in help-seeking behavior, willingness to disclose abuse, caregiving roles, and patterns of social dependence. Similarly, the association between lower educational attainment and increased EA risk may reflect broader socioeconomic inequalities, reduced access to information and support resources, and differences in health literacy rather than a direct causal relationship [26,28,29,36,37,39,41,44,46].

Several studies conducted during the COVID-19 pandemic reported higher levels of social isolation, psychological distress, economic difficulties, and reduced access to support services among older adults, all of which were associated with EA in the reviewed literature. Several studies conducted during the COVID-19 pandemic discussed public health restrictions, social distancing measures, and reduced access to healthcare and social services as factors potentially associated with increased social isolation among older adults. Simultaneously, economic instability, unemployment, psychological distress, and prolonged confinement within households were frequently discussed in the included studies as factors potentially associated with interpersonal tensions and caregiver burden. Importantly, the pandemic highlighted the critical role of social connectedness and access to support services in protecting vulnerable older adults [20,21,33,43,55].

The findings of this review have several important clinical, social, and public health implications. Across the included studies, low income, social isolation, disability, depressive symptoms, cognitive impairment, and functional dependence emerged as the most consistently reported factors associated with increased vulnerability to EA [25,26,27,28,29,30,31,32,34,35,36,37,39,40,41,42,44,45,46,47,48,49,50]. These findings indicate that preventive strategies should prioritize the early identification of older adults presenting cumulative psychosocial and health-related vulnerabilities rather than focusing exclusively on isolated risk factors. The reviewed evidence also highlights the importance of strengthening social support networks and reducing loneliness among older adults. Studies conducted during the COVID-19 pandemic reported associations between reduced social interaction, limited access to healthcare and support services, prolonged isolation, and increased vulnerability to EA. However, direct comparisons with pre-pandemic studies should be interpreted cautiously because of differences in study populations, settings, and assessment instruments [20,21,38,43,48]. Accordingly, interventions aimed at maintaining social participation, community engagement, and intergenerational connectedness may represent important protective strategies. Community-based initiatives, Universities of the Third Age, and programs promoting civic participation among older adults may help reduce social exclusion and strengthen informal support systems [21,56,57,58,59,60]. The findings additionally suggest the need for improved screening and monitoring procedures in both healthcare and social care settings. Older adults with chronic illness, cognitive impairment, depression, and functional limitations should be considered particularly vulnerable populations requiring regular assessment for potential abuse and neglect [26,29,41,45,46,50,51,52]. Increasing awareness and training among healthcare professionals, social workers, caregivers, and community organizations remain essential for improving early recognition of EA and facilitating timely intervention [40,57]. Furthermore, accessible psychological and social support services for both older adults and family caregivers, together with interdisciplinary collaboration across healthcare, social welfare, and community services, may strengthen prevention and response strategies for EA. In addition, the reviewed evidence suggests that broader structural determinants, including public policies, legal protection mechanisms, and the availability of healthcare and social service infrastructure, may influence both the occurrence and disclosure of elder abuse. These findings further support the need for coordinated system-level responses alongside individual- and community-based prevention strategies. Beyond individual and interpersonal risk factors, the reviewed evidence suggests that broader structural determinants—including public policies, legal protection mechanisms, healthcare accessibility, and the availability of social support services—may substantially influence both the occurrence and disclosure of elder abuse across different settings. These findings reinforce the view that elder abuse should be regarded as a complex public health challenge requiring coordinated responses at individual, community, institutional, and policy levels. In this broader context, the term “overlooked pandemic” reflects not only the worldwide occurrence of elder abuse but also its persistent under-recognition, underreporting, and insufficient prioritization within healthcare systems and public policy [21,26,41,43,50,51,52,53,54,55,56,57,58,59,60,61].

## 5. Limitations and Strengths of the Integrative Review

Several limitations should be considered when interpreting the findings. First, substantial methodological heterogeneity across study designs, populations, definitions of EA, assessment instruments, and analytical approaches limited direct comparability of the included studies and precluded meta-analysis. Second, cultural, socioeconomic, and healthcare differences across countries may have influenced both the occurrence and reporting of EA. Third, although the review followed PRISMA recommendations, complete database-specific search histories and exact search dates were not prospectively archived; however, reconstructed search strategies are provided in the Supplementary Materials. Google Scholar was used as a supplementary search source, which may have introduced additional variability in the retrieved records. Fourth, only English-language studies published between 2010 and 2022 were included, which may have introduced language bias and limits the review to evidence available during the pre-pandemic and pandemic periods. Fifth, the exclusion of disease-specific populations may limit the generalisability of the findings to some high-risk clinical groups. Finally, two included studies were authored by members of the research team; however, they met the same eligibility criteria as all other studies and were interpreted using identical methodological standards without preferential weighting.

This integrative review provides a comprehensive synthesis of international evidence on the prevalence, forms, and risk factors of elder abuse (EA) before and during the COVID-19 pandemic. By including studies conducted in community, clinical, institutional, and shelter-home settings, it offers a broad overview of EA across diverse cultural and healthcare contexts. To enhance transparency and support interpretation of the findings, a simplified methodological appraisal informed by selected domains of the JBI critical appraisal framework was performed (Supplementary Table S3). Although this appraisal was not intended to provide a formal study-level risk-of-bias assessment, it offered a structured overview of the methodological characteristics of the included studies.

## 6. Conclusions

This integrative review provides a comprehensive synthesis of the available evidence on the prevalence, forms, and risk factors of elder abuse (EA) among older adults before and during the COVID-19 pandemic. Despite considerable methodological and contextual heterogeneity across the included studies, low socioeconomic status, social isolation, disability, cognitive impairment, functional dependence, and depressive symptoms were consistently associated with increased vulnerability to EA. These findings highlight the importance of early identification of at-risk older adults and support the implementation of multidisciplinary prevention strategies involving healthcare, social care, and community services. Future research should prioritize standardized definitions, validated assessment instruments, and prospective study designs to improve the comparability of findings and strengthen the evidence base for the prevention and management of elder abuse.

## Figures and Tables

**Table 1:** Characteristics of the selected studies.

Author, Year	Study Design	Population	Age	Place	Measure
***During the COVID-19 pandemic***					
Koga et al., 2022 [38]	CS	18,236, 52.5% female	65–69 years—18.1% 70–74 years—30.1% 75–79 years—24.3% 80–84 years—17.1% 85≤—10.4%	mail survey	survey conducted by the Japan Gerontological Evaluation Study (JAGES)
Son and Cho, 2022 [43]	CS	3106, 55.7% female	65–74 years—61.2%, ≥75 years—38.8%	secondary data analysis of the 2020 Living Profiles of Older People Survey in Seoul	based on literature
Weissberger et al., 2022 [48]	DS	2410 calls were made across the two time period: Time 1 16 March 2018 to 15 March 2019: 1401 and Time 2 16 March 2020 to 15 March 2021: 1009 calls.	---	contacts made to the National Center on Elder Abuse (NCEA)	Elder abuse and its subtypes were defined based on a Center for Disease Control and Prevention (CDC) report
Du and Chen, 2021 [33]	CS	10,362, 57.1% female	65–69 years—44% 70–74 years—42% ≥75 years—14%	household questionnaire survey	the elderly abuse questionnaire in the “Third Survey on Chinese Women’s Social Status”
Chang and Levy, 2021 [20]	CS	897	≥60 years, mean age 68.9 ± 5.3	online survey	based on the literature (H-S/EAST and the Vulnerability to Abuse Screening Scale)
Filipska et al., 2021 [21]	CS	347, 55.9% female	65–70 years—46.7%, 71–85 years—34%, >85 years—19.3%	Department of Neurology and Department of Geriatrics	the Author-Designed Questionnaire; the Vulnerability to Abuse Screening Scale
***Before the COVID-19 pandemic***					
Ahnlund et al., 2020 [26]	CS	2851, 60.4% female	≥60 years	the survey online or postal questionnaire	based on the literature, HAD, PCL
Alraddadi, 2020 [27]	CS	446, 85.7% female	60–64 years—N = 209 (47%), 65–69 years—N= 72 (16%), 70–74 years—N = 59 (13%), 75 years—N = 106 (24%)	shelter homes	based on the literature
Dos Santos et al., 2020 [32]	CS	146, 43.8% female	≥60 years, mean age 73.35 ± 8.45	emergency care unit	H-S/EAST
Fang et al., 2020 [50]	PS	600, 55.8% male	≥60 years	geriatric departments	CTS2
Kulakçi, Altintas, and Korkmaz Aslan, 2020 [39]	CS	691	≥60, mean age—70.40 ± 5.04	family health centres	based on the literature
Sathya and Premkumar, 2020 [41]	CS	9852, 52.7% female	60–69 years—61.85%, 70–79 years—27.3%, ≥80 years—10.85%	household interviews	ADL, BKPAI, IADL
Sembiah et al., 2020 [42]	CS	246, 42% female	≥60 years, mean age; 71.82 ± 9.85	interviews conducted in a community setting	Actual Abuse Tool, ADL, IADL
Sudan et al., 2020 [44]	CS	300, Group 1—50% patients with psychiatric illnesses, Group 2—50% patients with somatic illness	≥65	the psychiatry OPD and OPDs of other departments with psychiatric and somatic illnesses	EASI
Filipska et al., 2019 [35]	CS	200, 56% female	≥60 years	geriatric departments	based on the literature
Yunus et al., 2017 [47]	CS	1648, 60.2% female	≥60 years	home-based interviews	questionnaire derived from the modified Conflict Tactic Scales (CTS)
Choi et al., 2018 [29]	CS	172, 69.2% female	≥65 years, age group: 65–69—N = 36 (20.9%), 70–79—N = 97 (56.4%), 80–89—N = 37 (21.5%), >90—N = 2 (36.0%)	community academies for the elderly, community mental health improvement centers	The assessment tool of the South Korea Elder Protection Agency, KGDS, IES-R-K
Torres-Castro et al., 2018 [45]	CS	487, 80.1% female	≥60 years, mean age-73.2 ± 8.0	household interviews	GMS, Frailty Phenotype
Hosseinkhan et al., 2017 [37]	CS	683, 54.8% female	>60 years, mean age 68.5 ± 7.6	health centers	H-S/EAST
Anand, 2016 [28]	CS	1435, 52.2% female	≥60 years, age group: 60–69—N = 971 (66.3%); 70–79—N = 342 (24.6%); ≥80—N = 122 (9%)	household interviews	BKPAI
Giraldo-Rodríguez et al., 2015 [36]	CS	1089, 50.3% female	≥60 yeas	household interviews	based on the literature
Chokkanathan, 2014 [30]	CS	902	>60 years	household interviews	CTS
Edirisinghe et al., 2014 [34]	CS	530, 65% female	≥60 years, mean age 68.5	hospital	H/S EAST
Abdel Rahman and El Gaafary, 2012 [25]	CS	1106, 53.2% female	≥60 years	household interviews	Actual Abuse Tool, ADL, EAI, GDS-15, Questionnaire to elicit elder abuse
Dong et al., 2012 [49]	PS	4627, 64.4% female	≥65 years	household interviews	ADL, Chicago Elder Self-Neglect Scale, MMSE
Naughton et al., 2012 [40]	CS	2021, 55% female	≥60 years, mean age-74 ± 6.6	household interviews	CTS, ADL
Wu et al., 2012 [46]	CS	2000, 40.1% female	≥60, mean age-68.8 ± 6.6	household interviews	H-S/EAST, VASS, GDS-15
Chompunud et al., 2010 [31]	CS	233, 73.4% female	≥60 years, mean age 69.7	household interviews	DCEA, IGSEA, EBA, FMRAQ, FRS

Note. ADL = Activities of Daily Living; BKPAI= Building a Knowledge Base on Population Ageing in India; CDC = Centers for Disease Control and Prevention; CS = cross-sectional study; CTS = Conflict Tactics Scale; CTS2 = Revised Conflict Tactics Scales; DCEA = Diagnostic Criteria for Elder Abuse; DS = descriptive study; EAI = Elder Assessment Instrument; EASI = Elder Abuse Suspicion Index; EBA = Elder’s Behavior Assessment; FMRAQ = Family Member at Risk Abuse Questionnaire; FRS = Family Relationship Scale;GDS-15 = 15-item Geriatric Depression Scale; GMS = Geriatric Mistreatment Scale; HAD = Hospital Anxiety and Depression Scale; H-S/EAST = Hwalek–Sengstock Elder Abuse Screening Test; IADL = Instrumental Activities of Daily Living; IES-R-K = Impact of Event Scale-Revised Korean version; IGSEA = Interview Guideline for Screening for Elder Abuse; JAGES = Japan Gerontological Evaluation Study; KGDS = Korean Geriatric Depression Scale; MMSE = Mini-Mental State Examination; N/A = not applicable; PCL = PTSD Checklist; PS = prospective study; PTSD = post-traumatic stress disorder; VASS = Vulnerability to Abuse Screening Scale.

## Data Availability

No new data were created or analyzed in this study. Data sharing is not applicable for this article.

## Supplementary Materials

The following supporting information can be downloaded at: https://www.mdpi.com/article/10.3390/nursrep16070249/s1, Table S1: Search strategies used in the integrative review; Table S2: Potential Sources of Heterogeneity Across Included Studies; Table S3: Simplified methodological appraisal of included studies; Table S4: Detailed risk factors of the analyzed scientific studies.

## Public Involvement Statement

No public involvement in any aspect of this research.

## Guidelines and Standards Statement

This study was conducted as an integrative review following the methodological framework of Whittemore and Knafl. PRISMA 2020 recommendations were used to support transparent reporting of the review process.
