# Supplementary material for: Behind Closed Doors: Elder Abuse as an Overlooked Pandemic of Modern Times
Source: Nurs Rep. 2026 Jul 17;16(7):249. doi: 10.3390/nursrep16070249 (PMC13414507; doi:10.3390/nursrep16070249)
Supplement: Supplementary file 1 [file nursrep-16-00249-s001.zip › nursrep-4287231-supplementary.pdf]

Table S1. Search strategies used in the integrative review.

Database	Search Strategy
PubMed	("elder abuse" OR "elder neglect" OR "elder mistreatment" OR violence OR "financial exploitation" OR "physical abuse" OR "psychological abuse" OR "emotional abuse" OR "sexual abuse") AND ("older adults" OR "older people" OR elderly OR seniors OR aged [MeSH Terms]) AND (prevalence OR incidence OR epidemiology OR "risk factors" OR perpetrators OR victims). **Filters:** English; Humans; Publication date: 2010/01/01–2022/12/31
Scopus	TITLE-ABS-KEY ("elder abuse" OR "elder neglect" OR "elder mistreatment" OR violence OR "financial exploitation" OR "physical abuse" OR "psychological abuse" OR "emotional abuse" OR "sexual abuse") AND ("older adults" OR "older people" OR elderly OR seniors OR aged) AND (prevalence OR incidence OR epidemiology OR "risk factors"). Limits: Document type: Article; Language: English; Years: 2010–2022
Google Scholar	"elder abuse" OR "elder neglect" AND "older adults" AND prevalence OR risk factors. The first pages of results sorted by relevance were screened.

For pandemic-related studies, the search was supplemented with the terms "COVID-19", "pandemic", and "coronavirus".

Table S2. Potential Sources of Heterogeneity Across Included Studies.

Source of heterogeneity	Examples from included studies	Examples identified in review	Potential impact on prevalence estimates
Study setting	Community, hospital/geriatric departments, emergency units, health centres, shelter homes, national surveys	Community studies reported prevalence from 2.2% to 45%; shelter-home studies up to 81.2%	Different exposure to abuse, reporting practices, and vulnerability profiles may substantially affect prevalence estimates.
Assessment instruments	H-S/EAST, VASS, CTS/CTS2, EASI, Actual Abuse Tool, GMS, author-designed questionnaires	More than 10 different instruments were used across studies	Differences in sensitivity, specificity, and operationalization of abuse may influence case identification.
Operational definition of elder abuse	Physical, psychological, financial, sexual abuse, neglect, self-neglect, mixed definitions	Some studies assessed overall EA, others focused on selected abuse types	Variation in definitions limits direct comparability of prevalence estimates.
Population characteristics	Community-dwelling older adults, clinical populations, institutionalized older adults	Mean age, sex distribution, disability burden, and health status varied considerably	Different risk profiles may influence observed prevalence and associated factors.
Geographical and cultural context	Asia, Europe, North America, Middle East, Latin America	Studies conducted in Japan, Korea, China, India, Saudi Arabia, Sweden, Mexico, Ireland, USA and others	Cultural norms, family structures, reporting behaviors, and social support systems may affect both occurrence and disclosure of abuse.
Pandemic vs. pre-pandemic period	Studies conducted before COVID-19 and during COVID-19	Pandemic studies reported prevalence from 1.6% to 44.7%	Differences in social isolation, healthcare access, and public health restrictions may influence reported abuse patterns.
Study design	Predominantly cross-sectional studies with a small number of prospective and descriptive studies	Most included studies were cross-sectional	Differences in study design may affect identification and interpretation of risk factors.

Note. This table summarizes major methodological and contextual factors identified across the included studies that may contribute to heterogeneity in prevalence estimates and reported risk factors.

Table S3. Simplified methodological appraisal of included studies

Note. This table presents a simplified methodological appraisal informed by key JBI appraisal domains for observational studies, including study design, sample adequacy, measurement approach, and reporting of statistical analyses. It should be interpreted as a screening-level assessment rather than a complete formal JBI critical appraisal.

Study	Study design	Sample size	Measurement approach	Key methodological considerations	Overall appraisal	Interpretation
Koga et al., 2022 [38]	CS	18,236	JAGES mail survey	Large population-based sample; clearly reported statistical estimates	High	Findings provide relatively stronger descriptive evidence within the limits of the observational design.
Son and Cho, 2022 [43]	CS	3,106	Secondary data analysis; literature-based tool	Large sample; multivariable risk estimates reported; measurement based on prior literature	High	Findings provide relatively stronger descriptive evidence within the limits of the observational design.
Weissberger et al., 2022 [47]	DS	2,410 calls	NCEA resource line; CDC-based definitions	Large administrative/contact dataset; descriptive design limits causal interpretation	Moderate	Findings should be interpreted with caution due to limitations such as smaller sample size, clinical/specific setting, or measurement variability.
Du and Chen, 2021 [33]	CS	10,362	Household questionnaire survey	Large household survey; multiple risk factors and statistical estimates reported	High	Findings provide relatively stronger descriptive evidence within the limits of the

						observational design.
Chang and Levy, 2021 [20]	CS	897	Online survey; H-S/EAST and VASS-based items	Moderate sample; established instruments partly used; pandemic comparison reported	Moderate	Findings should be interpreted with caution due to limitations such as smaller sample size, clinical/specific setting, or measurement variability.
Filipska et al., 2021 [21]	CS	347	Author-designed questionnaire; VASS	Moderate sample; VASS included; author-designed questionnaire may limit measurement comparability	Moderate	Findings should be interpreted with caution due to limitations such as smaller sample size, clinical/specific setting, or measurement variability.
Ahnlund et al., 2020 [26]	CS	2,851	Online/postal questionnaire; literature-based tool, HAD, PCL	Large sample; validated psychological measures used; statistical estimates reported	High	Findings provide relatively stronger descriptive evidence within the limits of the observational design.
Alraddadi, 2020 [27]	CS	446	Shelter homes; literature-based tool	Specific shelter-home population; moderate sample; measurement based on prior literature	Moderate	Findings should be interpreted with caution due to limitations such as smaller sample size, clinical/specific setting, or measurement variability.

Dos Santos et al., 2020 [32]	CS	146	Emergency care unit; H-S/EAST	Small clinical sample; validated screening tool used; limited generalisability	Moderate	Findings should be interpreted with caution due to limitations such as smaller sample size, clinical/specific setting, or measurement variability.
Fang et al., 2020 [50]	PS	600	Geriatric departments; CTS2	Prospective design; validated instrument; clinical population	High	Findings provide relatively stronger descriptive evidence within the limits of the observational design.
Kulakçi Altintas and Korkmaz Aslan, 2020 [39]	CS	691	Family health centres; literature-based tool	Moderate sample; risk estimates reported; measurement based on literature	Moderate	Findings should be interpreted with caution due to limitations such as smaller sample size, clinical/specific setting, or measurement variability.
Sathya and Premkumar, 2020 [41]	CS	9,852	Household interviews; ADL, BKPAI, IADL	Very large sample; standardized functional measures; statistical estimates reported	High	Findings provide relatively stronger descriptive evidence within the limits of the observational design.
Sembiah et al., 2020 [42]	CS	246	Community interviews; Actual Abuse Tool, ADL, IADL	Moderate/small community sample; multiple standardized tools;	Moderate	Findings should be interpreted with caution due to

				statistical estimates reported		limitations such as smaller sample size, clinical/specific setting, or measurement variability.
Sudan et al., 2020 [44]	CS	300	Psychiatry and other OPDs; EASI	Clinical sample; validated screening tool; limited population generalisability	Moderate	Findings should be interpreted with caution due to limitations such as smaller sample size, clinical/specific setting, or measurement variability.
Filipska et al., 2019 [35]	CS	200	Geriatric departments; literature-based tool	Small clinical sample; measurement based on literature; statistical estimates reported	Moderate	Findings should be interpreted with caution due to limitations such as smaller sample size, clinical/specific setting, or measurement variability.
Yunus et al., 2017 [47]	CS	1,648	population-based cross-sectional study	large sample; home-based interviews; validated questionnaires (modified CTS for elder abuse)	High	Findings provide descriptive evidence of an association, although temporal and causal relationships cannot be determined.
Choi et al., 2018 [29]	CS	172	Community centres; Korean Elder Protection Agency tool,	Small sample; validated psychological measures; statistical estimates reported	Moderate	Findings should be interpreted with caution due to

			KGDS, IES-R-K			limitations such as smaller sample size, clinical/specific setting, or measurement variability.
Torres-Castro et al., 2018 [45]	CS	487	Household interviews; GMS, Frailty Phenotype	Moderate sample; validated mistreatment/frailty measures; statistical reporting partly descriptive	Moderate	Findings should be interpreted with caution due to limitations such as smaller sample size, clinical/specific setting, or measurement variability.
Hosseinkhani et al., 2017 [37]	CS	683	Health centers; H-S/EAST	Moderate sample; validated screening tool; statistical estimates reported	Moderate	Findings should be interpreted with caution due to limitations such as smaller sample size, clinical/specific setting, or measurement variability.
Anand, 2016 [28]	CS	1,435	Household interviews; BKPAI	Large sample; standardized ageing dataset; statistical estimates reported	High	Findings provide relatively stronger descriptive evidence within the limits of the observational design.
Giraldo-Rodríguez et al., 2015 [36]	CS	1,089	Household interviews; literature-based tool	Large sample; risk estimates reported; measurement based on literature	High	Findings provide relatively stronger descriptive evidence within the

						limits of the observational design.
Chokkanathan, 2014 [30]	CS	902	Household interviews; CTS	Moderate/large sample; validated instrument; statistical estimates reported	High	Findings provide relatively stronger descriptive evidence within the limits of the observational design.
Edirisinghe et al., 2014 [34]	CS	530	Hospital; H/S EAST	Moderate clinical sample; validated screening tool; limited statistical detail	Moderate	Findings should be interpreted with caution due to limitations such as smaller sample size, clinical/specific setting, or measurement variability.
Abdel Rahman and El Gaafary, 2012 [25]	CS	1,106	Household interviews; Actual Abuse Tool, ADL, EAI, GDS-15	Large sample; multiple standardized measures; statistical estimates reported	High	Findings provide relatively stronger descriptive evidence within the limits of the observational design.
Dong et al., 2012 [49]	PS	4,627	Household interviews; ADL, Chicago Elder Self-Neglect Scale, MMSE	Prospective design; large sample; standardized measures	High	Findings provide relatively stronger descriptive evidence within the limits of the observational design.
Naughton et al., 2012 [40]	CS	2,021	Household interviews; CTS, ADL	Large national survey; validated instrument and functional measure;	High	Findings provide relatively stronger descriptive

				statistical estimates reported		evidence within the limits of the observational design.
Wu et al., 2012 [46]	CS	2,000	Household interviews; H-S/EAST, VASS, GDS-15	Large sample; multiple validated measures; statistical estimates reported	High	Findings provide relatively stronger descriptive evidence within the limits of the observational design.
Chompunud et al., 2010 [31]	CS	233	Household interviews; DCEA, IGSEA, EBA, FMRAQ, FRS	Small sample; multiple elder-abuse-related instruments; wide confidence intervals reported	Moderate	Findings should be interpreted with caution due to limitations such as smaller sample size, clinical/specific setting, or measurement variability.

CS = cross-sectional study; DS = descriptive study; PS = prospective study; JBI = Joanna Briggs Institute; EA = elder abuse.

Table S4. Detailed risk factors of the analyzed scientific studies

Author, year	*Risk factors*
Koga et al., 2022 [38]	- refrainment from various daily activities during the COVID-19 pandemic<br>- reduced interaction with neighbors<br>- reduced grocery shopping
Son and Cho, 2022 [43]	- age<br>- type of household<br>- self-rated health<br>- cognitive impairments<br>- smoking<br>- depressive symptoms<br>- frequency of alcohol consumption<br>- number of comorbidities<br>- number of family members<br>- social isolation due to COVID-19
Weissberger et al, 2022 [48]	---
Du and Chen, 2021 [33]	- age

	- gender<br>- marital status<br>- education level<br>- income<br>- number of children<br>- health condition<br>- cognitive ability<br>- social frequency<br>- religious belief<br>- income
Chang and Levy, 2021 [20]	- sense of community<br>- physical distancing<br>- financial strain<br>- age<br>- self-rated health
Filipska et al., 2021 [21]	- gender<br>- age<br>- equivalent family income<br>- place of residence<br>- chronic disease<br>- loneliness<br>- participation in family decisions<br>- relationship with the family<br>- depression<br>- COVID–19 in the past<br>- Activities of Daily Living (ADL)
Ahnlud et al., 2020 [26]	- education<br>- gender<br>- income<br>-physical health<br>- psychological health<br>- PTSD
Alraddadi, 2020 [27]	- age<br>- chronic disease<br>- gender<br>- having children<br>- income<br>- marital status
Dos Santos et al., 2020 [32]	- age<br>- frailt syndrome<br>- number of children,<br>- income
Fang et al., 2020 [49]	- cognitive impairment<br>- depression<br>- neuropsychiatric symptoms
KulakçiAltintas¸and Korkmaz Aslan, 2020 [39]	- age<br>- education<br>- family relationship<br>- income
Sathya and Premkumar, 2020 [41]	- >ADLs<br>- disability

	- education<br>- >IADLs<br>- income<br>- marital status
Sembiah et al., 2020 [42]	- age<br>- chronic diseases<br>- depression<br>- disability<br>- gender<br>- income<br>- number of children<br>- type of family
Sudan et al., 2020 [44]	- education<br>- gender<br>- marital status<br>- psychiatric illness<br>- somatic illness
Filipska et al., 2019 [35]	- age<br>- gender<br>- income<br>- place of residence
Yunus et al., 2017 [47]	- income,<br>- GDS score
Choi et al., 2018 [29]	- depression<br>- education<br>- marital status<br>- PTSD
Torres-Castro et al., 2018 [45]	- anxiety<br>- depression<br>- > Charlson Comorbidity Index scores<br>- > frequency of frailty,<br>- urinary and fecal incontinence
Hosseinkhan et al., 2017 [37]	- age<br>- education<br>- income<br>- place of resident

Anand, 2016 [28]	- age<br>- disability<br>-education<br>- income<br>- living with family
Giraldo-Rodríguez et al., 2015 [36]	- age<br>- education<br>- emotional symptoms<br>- employment<br>- past hospitalization, proportion that received other social support<br>- place of residence,<br>- proportion with a primary caregiver<br>- self-rated health, number of disabilities
Chokkanathan, 2014 [30]	- age<br>- dependent<br>- employments<br>- gender
Edirisinghe et al., 2014 [34]	- marital status
Abdel Rahman and El Gaafary, 2012 [25]	- age<br>- dependent<br>- employment<br>- gender<br>- number of children<br>- marital status<br>- mental health<br>- physical health
Dong et al., 2012 [49]	- physical function
Naughton et al., 2012 [40]	- income<br>- mental health<br>- physical health<br>- social support

Wu et al., 2012 [46]	- chronic disease<br>- depression<br>- education<br>- income<br>- living alone<br>- marital status<br>- physical disability
Chompunud et al., 2010 [31]	- family relationship<br>- gender<br>- income<br>- perception of health and personal health
